# Cost-utility of cochlear implantation in single-sided deafness and asymmetric hearing loss: results of a randomized controlled trial

**DOI:** 10.1007/s10198-024-01740-9

**Published:** 2024-11-20

**Authors:** Mathieu Marx, Michaël Mounié, Isabelle Mosnier, Frédéric Venail, Michel Mondain, Alain Uziel, David Bakhos, Emmanuel Lescanne, Yann N’Guyen, Daniele Bernardeschi, Olivier Sterkers, Benoit Godey, Gwenaëlle Creff, Sébastien Schmerber, Nicolas-Xavier Bonne, Christophe Vincent, Bernard Fraysse, Olivier Deguine, Nadège Costa

**Affiliations:** 1 Service d’Otologie, Otoneurologie et ORL Pédiatrique, Hôpital Pierre-Paul Riquet, CHU Toulouse Purpan, Place du Dr Baylac, Toulouse Cedex, 31059 France; 2 Brain & Cognition Research Centre, UMR 5549, Université Toulouse III, Toulouse, France; 3 Unité d’Evaluation Médico-Economique, CHU Toulouse, Toulouse, France; 4 Unité Fonctionnelle Implants Auditifs, ORL, GH Pitié-Salpêtrière, AP-HP Sorbonne Université, Paris, France; 5 Technologies et Thérapie Génique Pour la Surdité, Institut de L’Audition, Institut Pasteur / Inserm/ Université Paris Cité, Paris, France; 6 Service d’ORL, Hôpital Gui de Chauliac, CHU Montpellier, Montpellier, France; 7 Service d’ORL, Hôpital Bretonneau, CHU Tours, Tours, France; 8 Service d’ORL, Hôpital Pontchaillou, CHU Rennes, Rennes, France; 9 Service d’ORL, CHU Grenoble, Grenoble, France; 10 Université Lille, Inserm, CHU Lille, Service d’ORL, U1192 – PRISM, Lille, France; 11 Service d’ORL, Hôpital Salengro, CHU Lille, Lille, France

**Keywords:** Single-sided deafness, Asymmetric hearing loss, ICUR, Cochlear implant, Tinnitus, I18 - Health policy, Regulation, Public health

## Abstract

**Objectives:**

To determine the Incremental Cost-Utility Ratio (ICUR) of cochlear implantation in the treatment of adult patients with single-sided deafness (SSD) and asymmetric hearing loss (AHL).

**Methods:**

This prospective multicenter pragmatic study including a randomized controlled trial (RCT) enrolled 155 subjects with SSD or AHL. Subjects chose a treatment option between: abstention, Contralateral Routing Of the Signal hearing aids, Bone Conduction Device or Cochlear Implant (CI). Participants who opted for CI were then randomized between two arms: “immediate CI” where the cochlear implantation was performed within one month and “initial observation” where subjects were first observed. The ICUR of CI was determined at 6 months follow-up by comparing the two arms. Utility was measured using EuroQoL- 5 dimensions (EQ-5D), to calculate the gain in Quality-Adjusted Life Years (QALY). Individual costs were extracted from the French National Health Insurance database. A Markovian MultiState (MMS) model assessed the ICUR evolution over the lifetime horizon.

**Results:**

Among the 155 included participants, 51 opted for a CI and were randomized. For a 6 months follow-up period, the ICUR was €422,279/QALY gained after CI. Using the MMS model, the ICUR of CI decreased to €57,561/QALY at 10 years follow-up, €38,006/QALY at 20 years, and dropped to €26,715 at 50 years. In the participants with severe tinnitus, mean ICUR was €31,105/QALY at 10 years.

**Conclusions:**

CI can be considered as an efficient treatment in SSD and AHL from 20 years follow-up in the global population, and before 10 years follow-up in patients with severe associated tinnitus.

**Supplementary Information:**

The online version contains supplementary material available at 10.1007/s10198-024-01740-9.

## Introduction

Over the past three decades, cochlear implantation has allowed restoring hearing in more than one million individuals affected with bilateral severe-to-profound deafness worldwide [1]. Its outcomes on speech recognition and oral communication generate significant improvements in quality of life, whether it is measured using generic or specific to hearing questionnaires (see [2, 3] for systematic review). Likewise, cochlear implantation has been established as an efficient treatment for bilateral severe-to-profound deafness, from various healthcare systems perspectives, in adults [4] as in children [5].

In single-sided deafness (SSD) and asymmetric hearing loss (AHL), speech understanding in quiet environments is usually preserved and most hearing deficits are usually related to the disruption of binaural hearing, i.e. integration of sound signals incoming to each ear [6, 7]. With an estimated prevalence of 0.14% in adult population [8], such deficits have been shown to alter quality of life (QoL) mean scores through psychological and social consequences [9, 10]. In SSD, the better ear has normal or near-to-normal hearing thresholds and there is a profound hearing loss in the poor ear [11]. In AHL, the better ear shows mild-to moderate hearing loss while profound hearing loss affects the other ear [11]. Typically, these conditions lead to difficulties for understanding speech in noisy environments and to deficits for sound localization. However, the functional limitations may vary remarkably from a slight deficit in localization to significant impairment in speech recognition in noise, associated with debilitating tinnitus, with a significant inter-individual variability [12]. To address this handicap heterogeneity, the invasiveness of the therapeutic options for this condition gradually increased through last decades, from Contralateral Routing of the Signal (CROS) hearing aids in the mid-1960s to bone conduction devices (BCD) in the 2000s, and cochlear implants in more recent years. The first two options (CROS and BCD) allow the transfer of the sound incoming to the poor ear toward the better ear, using BlueTooth connected hearing aids (CROS) or the sound conductive properties of the skull with a BCD. Among all options, the cochlear implant (CI) is the only treatment which provides an auditory stimulation of the poor ear. The results of cochlear implantation are quite consistent on tinnitus when associated with single-sided deafness or asymmetric hearing loss, with a significant reduction of tinnitus severity in more than 80% of cases (see Levy et al. for review [13]). To a lesser extent and to varying degrees, it may improve speech recognition in noisy environments and spatial hearing [14, 15]. However, the cost of the device itself and the afferent procedures in the case of unilateral cochlear implantation vary between €29,000 to 44,000 depending on the country [16, 17], which legitimately raises the question of cost-effectiveness and reimbursement of CI in this new indication. This question remains unrecognized because scarce and recent literature on that topic only relied on models applied to extrapolated clinical data and expert opinions [18, 19].

The present article reports the health-economic results of a national multicenter prospective study designed to determine the incremental cost-utility ratio (ICUR) of CI in SSD and AHL, in a randomized controlled trial conducted after failure of CROS hearing aids and BCDs trials. Treatment choice among all options (CROS, BCD, CI, observation) and related efficacy on hearing outcomes and quality of life have previously been described [12, 20]. The ICUR of CI was then assessed in a lifetime horizon using a multi-state Markovian model.

## Methods

All of the authorizations required by French legislation were obtained for this study, which was approved by the Ethics Committee on May 2014 under the reference CPP14-020/2014-A00533-44. All the patients included have received verbal and written information and an informed consent form was signed before starting the trial.

### Study design and oversight

This national multicenter, pragmatic study was conducted in 7 tertiary referral centers in France, between November 2014 and December 2018. It was divided into 2 parts (see Marx et al. for details [21]) to reflect routine clinical management of subjects with SSD or AHL. Such subjects usually first try CROS hearing aids and BCDs before considering a possible cochlear implantation. Therefore, the first part of this trial was a prospective, descriptive observational cohort study, with a 6-month follow-up for SSD/AHL adult subjects who chose to be treated by CROS hearing aids, or by BCDs, or by CI and patients who declined all the options, after 2 consecutive trials of the CROS and BCDs. The second part was an open-label randomized controlled clinical trial for subjects with SSD/AHL who chose to be treated by the CI after failure of both CROS and BCD trials, in 2 parallel arms: cochlear implantation versus observation for 6 months. The subjects randomized in the “observation” arm could benefit from a cochlear implantation procedure once this 6-month observation initial period had ended. Finally, a Markov Multi State (MMS) model was used to simulate the cost-effectiveness of CI in comparison with observation over several time horizons up to a lifetime horizon (i.e. after 50 years).

### Participants

The trial enrolled adults older than 18 years, with SSD (better ear pure-tone average (PTA) better than 30 dB HL) or AHL (better ear PTA between 30 and 60 dB HL) documented using PTA on four frequencies (0.5, 1, 2 and 4 kHz) on pure-tone audiometry [11]. Patients with SSD/AHL due to a vestibular schwannoma or to major modifications of cochlear anatomy were excluded. As a pragmatic trial, and to account for the heterogeneity of this population seen in clinical practice, there was no inclusion criteria applied on the duration of deafness, the severity of a putative associated tinnitus, or PTA in the better ear (between 0 and 60 dB HL).

### Interventions and randomization procedure

All subjects chose one treatment option after two consecutive trials of CROS hearing aids and BCD (3 weeks for each trial). This choice was made between the 4 following options: abstention, CROS, BCD, and randomized for CI (RCI). The choice was mainly based on the subjective outcomes reported after the 2 trials but also guided by the auditory outcomes obtained with each device, the recommendations of the corresponding manufacturer, and the counselling of the physician [21].

In the group RCI, patients were randomized to a 6-month “observation” arm or an immediate cochlear implantation “CI” arm. The CI, supporting 12–22 intra-cochlear electrodes, was placed after the traditional surgical procedure. Four major companies were represented (Advanced Bionics^®^, Cochlear^®^, MedEl^®^, and Oticon Medical^®^). Randomization of CI versus initial observation in the RCI group was based on a 1:1 ratio and was stratified per center. The allocation sequence was randomly and automatically generated (Stata SE 11.2, ralloc procedure).

## Outcomes and follow-up

### Primary outcome measures

The primary outcome measure was the incremental cost-utility ratio (ICUR) of CI compared to observation for a 6-month follow-up period and modelled over the lifetime horizon. Utility was measured through preference-based health-related quality of life (0 indicates death, 1, perfect health with a meaningful clinically important difference of 0.05). It was assessed using EuroQol 5 Dimensions (EQ-5D), as recommended by the French Health Authority (*Haute Autorité de Santé*), and allowed the calculation of QALYs when multiplied by years gained. Indeed, EQ-5D is to date the only generic instrument recommended in France for cost-utility studies [22]. It includes a descriptive component and a Visual Analogue Scale (VAS) (EQ-VAS). The descriptive component is composed of 5 dimensions (mobility, autonomy, daily life activities, pain, and anxiety/depression) described by 3 levels. Utility considered in the ICUR assessment in each arm was the difference between utility at 6 months and utility at baseline in order to assess the gain or loss due to the intervention or the absence of intervention.

Cost assessment was performed first from the French National Health Insurance (FNHI) perspective using FNHI database [23]. Direct medical (i.e. inpatient stays, medical devices, outpatient care, drugs) and non-medical costs (i.e. transportation) as well as costs related to absence from the workplace due to illness (i.e. productivity lost) were considered and valued according to €2021 (see supplementary file 1). First, real-world expenses incurred in the management of patients were estimated over a 6-month period from the FNHI database. Finally, the cost of management was modeled over a lifetime horizon using: (1) expert opinion to define the type and quantity of care associated with SSD management every 6 months according to health states, and (2) FNHI tariffs to value the management. Inpatient stays were costed using the French framework for hospital activity pricing.

### Secondary outcome measures

The ICUR of CI compared to observation was further investigated using a mapping between EQ-5D and Health Utility Index version 3 (HUI-3). This mapping procedure was performed because it has been demonstrated that EQ-5D may not fully capture quality of life changes related to hearing modifications [24, 25]. Because HUI-3 includes two sensorial components (vision and hearing), it has been identified as the preferred instrument in the assessment of treatments for SSD and AHL [24]. The mapping procedure is detailed in supplementary file 2. The impact of tinnitus associated to SSD/AHL on the cost-effectiveness of CI was assessed by comparing ICURs (at 6-month follow-up and using Markovian model) in participants with severe tinnitus (VAS ≥ 6/10) or without severe tinnitus (VAS ≤ 5/10).

### Statistical analysis

Descriptive statistics were used to describe groups, characteristics and mean with 95% BCA bootstraps non-parametric confidence interval to assess the total cost and the utility.

Costs and utility missing data for the CI vs. observation comparison were imputed using multiple imputation and predictive mean matching method. Age, gender, comorbidity (0/1), tinnitus and baseline level quality of life were used as confounding factors.

**Fig. 1:**
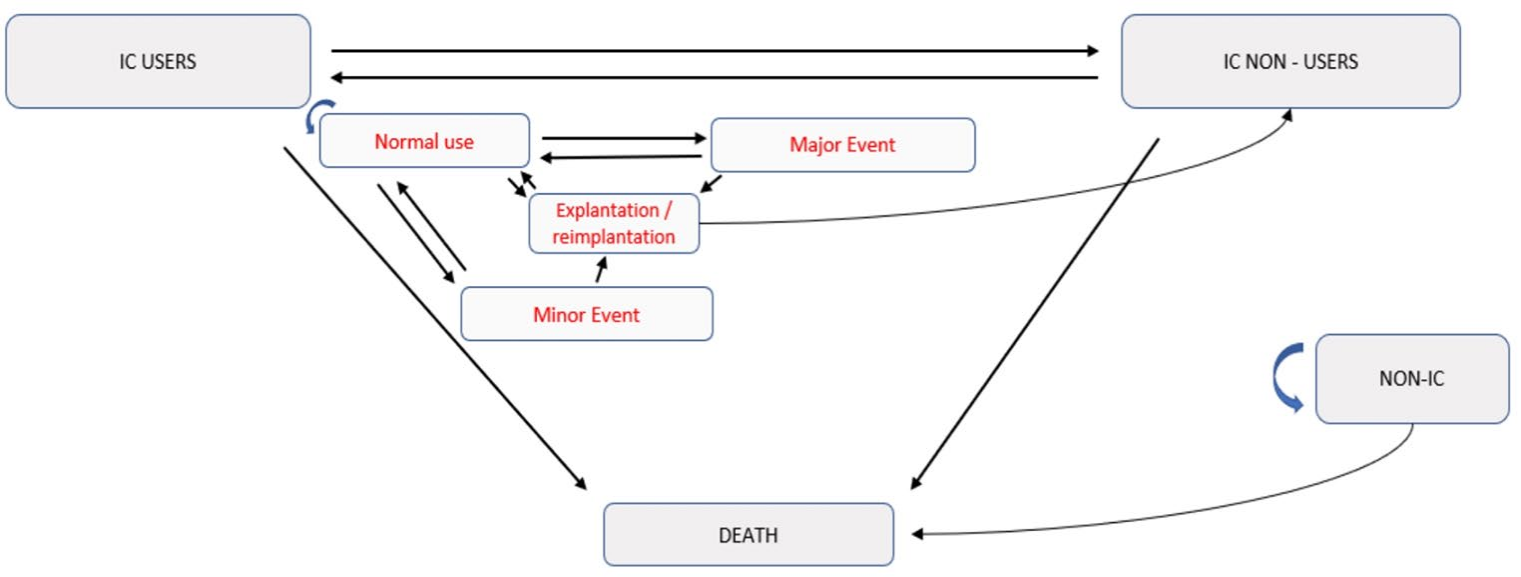
Multistatus Markovian model used to model lifetime evolution of the Incremental Cost-Utility Ratio of cochlear implant

An MMS model was used to model the ICUR evolution over lifetime horizon (see Fig. 1). The different status and transitions probabilities in this model were included based on the rates identified in literature with 1/ the rate of CI non-users in SSD/AHL [12, 20], 2/ the rates of major or minor events following cochlear implantation [26], 3/ the rate of explantation and reimplantation [27]. All the assumptions which have been made to build the model in addition to details on all states and the possible paths through the states are described in the supplementary files (supplementary file 3 and e-Table 1).

The model performed 1000 iterations on a putative cohort of 1000 individuals. A 6-month step time was selected to fit with the primary outcome. The MMS model was implemented according to different time horizons, from 10 to 50 years. The baseline case was characterized by a 45-year-old population to fit with the population in our data. Nevertheless, in addition to probabilistic sensitive analysis using replication to build a 95% confidence ellipse around the mean ICUR [28], several determinist sensitive analyses were implemented considering mean age equal to 20, 40 and 60 years old, the HUI3 mapped utility and depending on tinnitus or not (see Table 1 and supplementary file 4).

**Table 1:** Demographic and clinical characteristics of the 51 participants included in the randomized controlled trial

		CI	Initial Observation	
		*n* = 25	*n* = 26	*p*
Age	Mean (sd)	53,5 (12,7)	56,6 (9,9)	0.343
Gender, Male	n (%)	12 (48)	8 (30,8)	0.1599
Duration of deafness	< 1 year	6 (24)	6 (23,1)	0.881
	Between 1 and 5	8 (32)	7 (26,9)	
	> 5 years	11 (44)	13 (50)	
PTA good ear	Mean dB HL (sd)	27.6 (17.8)	30 (16.3)	
PTA poor ear	Mean dB HL (sd)	104.4 (20)	97.8 (19.7)	
SSD/AHL	n : SSD/AHL	12/13	12/14	
Severe tinnitus (VAS > 6/10)	n (%)	12 (48)	11 (42,3)	0.781
Etiology	Meniere’s disease	1 (4)	1 (3,8)	0.5326
	Labyrinthitis	1 (4)	2 (7,7)	0.4
	Labyrinthine trauma	3 (12)	0	0.5326
	Sudden sensorineural Hearing loss	10 (40)	12 (46,2)	0.994
	Unknown	10 (40)	11 (42.3)	0.994
Occupation	Managers and professionals	3 (12)	7 (26,9)	0.747
	Craft, services and sales workers	2 (8)	1 (3,8)	
	Clerical support workers	3 (12)	3 (11,5)	
	Technicians and associate professionals	14 (56)	12 (46,2)	
	Operators, asemblors	1 (4)	2 (7,7)	

## Results

### Patient characteristics

At the 7 study sites, 155 subjects were included in the trial with a 6-month follow-up visit for 138 of them (89%). Six participants withdrew their consent, 5 were lost to follow-up, 4 were excluded due to medical reasons, and the last 2 participants due to major protocol violations. After the two consecutive CROS and BCD trials, 75 subjects opted for CROS hearing aids, 18 for a BCD, 51 chose to be randomized for a CI and 11 eventually declined all treatment options. In the group of patients randomized for CI, 25 participants were allocated to the “CI arm” and 26 to the “initial observation arm” (see flowchart of the study in supplementary file 5). No baseline variable was significantly different between the two randomized arms (see Table 2) but some differences (gender distribution, comorbidities, initial EQ-5D scores) required statistical adjustments.

**Table 2:** Summary of the different sensitivity analyses performed as a function of follow-up duration, QoL indices which were used (EQ-5D or HUI3), and the presence of a severe associated tinnitus (VAS > 6/10)

	Scenarios / follow-up (years)	10	20	30	40	50
**Cost (2021€)**						
	Base case	31,515	40,467	46,667	51,928	53,903
Utility	HUI3	31,504	40,470	46,670	51,931	53,906
Age	20	31,561	40,907	47,978	55,355	61,153
	40	31,530	40,650	47,162	53,171	56,428
	60	31,302	39,357	43,618	44,848	44,899
Severe Tinnitus (VAS > 6/10)	Y	31,505	40,459	46,658	51,909	53,885
	N	31,498	40,456	46,652	51,906	53,878
**QALY**						
	Base case	0.550	1.074	1.543	1.919	2.109
Utility	HUI3	0.837	1.634	2.353	2.929	3.214
Age	20	0.556	1.104	1.648	2.178	2.679
	40	0.554	1.088	1.586	2.016	2.305
	60	0.537	1.001	1.312	1.406	1.411
Severe Tinnitus	Y	0.613	1.192	1.713	2.126	2.331
	N	0.442	0.862	1.242	1.545	1.699
**ICUR (€/QALY)**						
	Base case	57,561	38,006	30,764	27,907	26,715
Utility	HUI3	37,686	24,835	19,921	17,857	16,945
Age	20	56,851	37,104	29,181	25,513	22,986
	40	57,111	37,581	30,085	26,900	25,257
	60	59,072	40,540	35,284	34,552	34,531
Severe Tinnitus	Y	51,539	34,179	27,606	24,982	23,889
	N	71,736	47,585	38,636	35,263	34,524

### Primary outcome measure

**Fig. 2:**
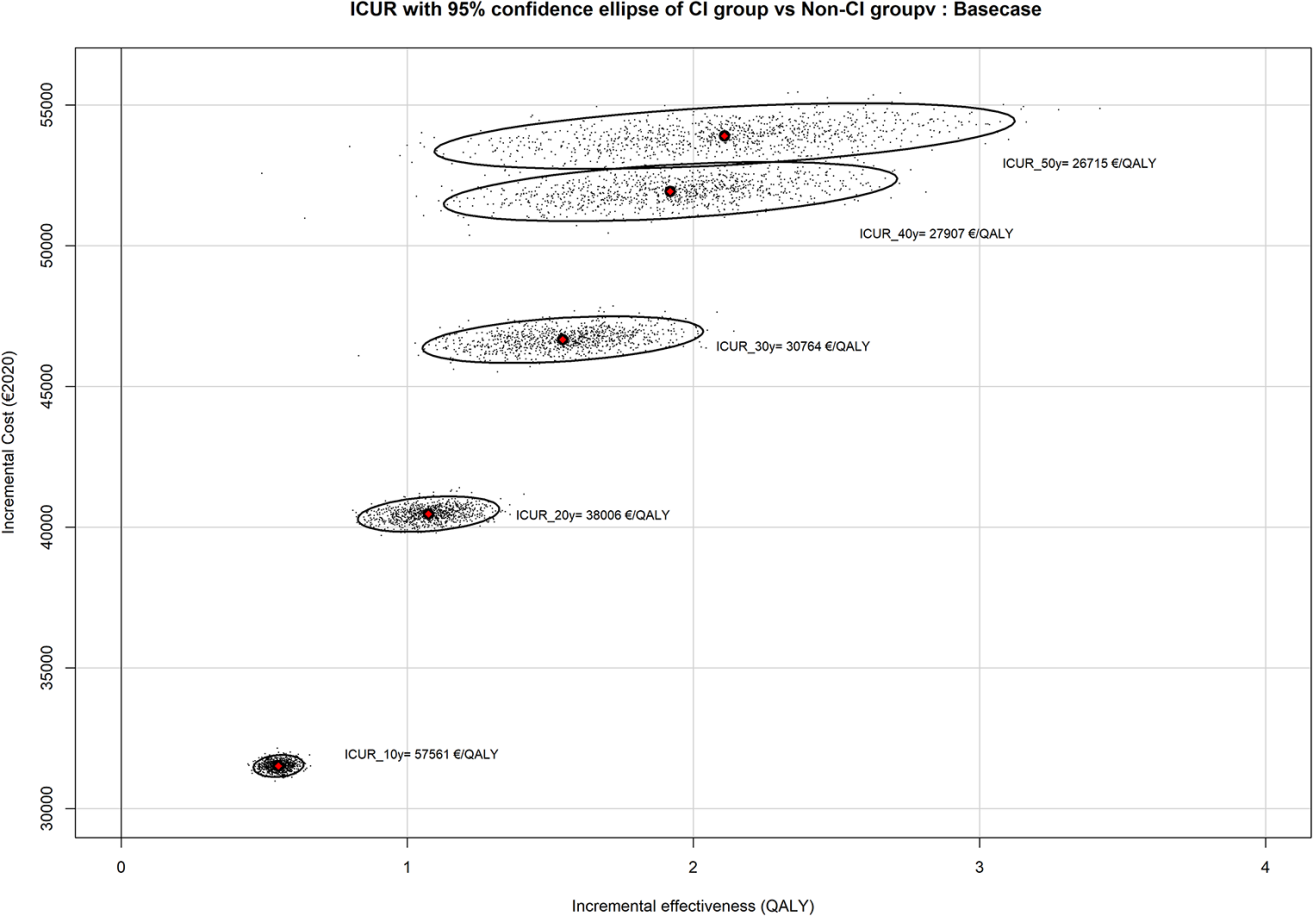
Confidence ellipses for ICUR at different follow-up intervals

Once imputed and adjusted, mean costs in the CI arm were €27,994 [27,563 − 28,481] and €1686 [1510–1916] in the initial observation arm. Six months after, utility was measured with a mean gain of 0.0459 QALY [0.01195–0.0702] in the CI arm, and a mean value of -0.0164 [-0.0425-0.0074] in the initial observation arm. Altogether, the mean differences between the two arms in terms of cost were €26,308€ and 0.0623 in terms of utility. Based on these differences, the ICUR was €422,279/QALY gained after CI for SSD and AHL, for a 6-month follow-up period. According to the MMS modeling within the base case scenario, mean ICUR was €57,561/QALY at 10-year follow-up, €38,006/QALY at 20 years, and dropped to €26,715 at 50 years (Fig. 2).

### Secondary outcome measures

#### HUI3 utility after mapping procedure

At 6-month follow-up, the comparison between CI arm and observation arm showed that mean incremental QALY was 0.0945 and the ICUR was then €278,392/QALY. After application of the MMS model, ICUR was equal to €37,686/QALY at 10-year follow-up, €24,835/QALY at 20 years, and decreased to €16,945/QALY at 50 years (supplementary file 3).

#### Participants with severe tinnitus (VAS > 6/10)

**Fig. 3:**
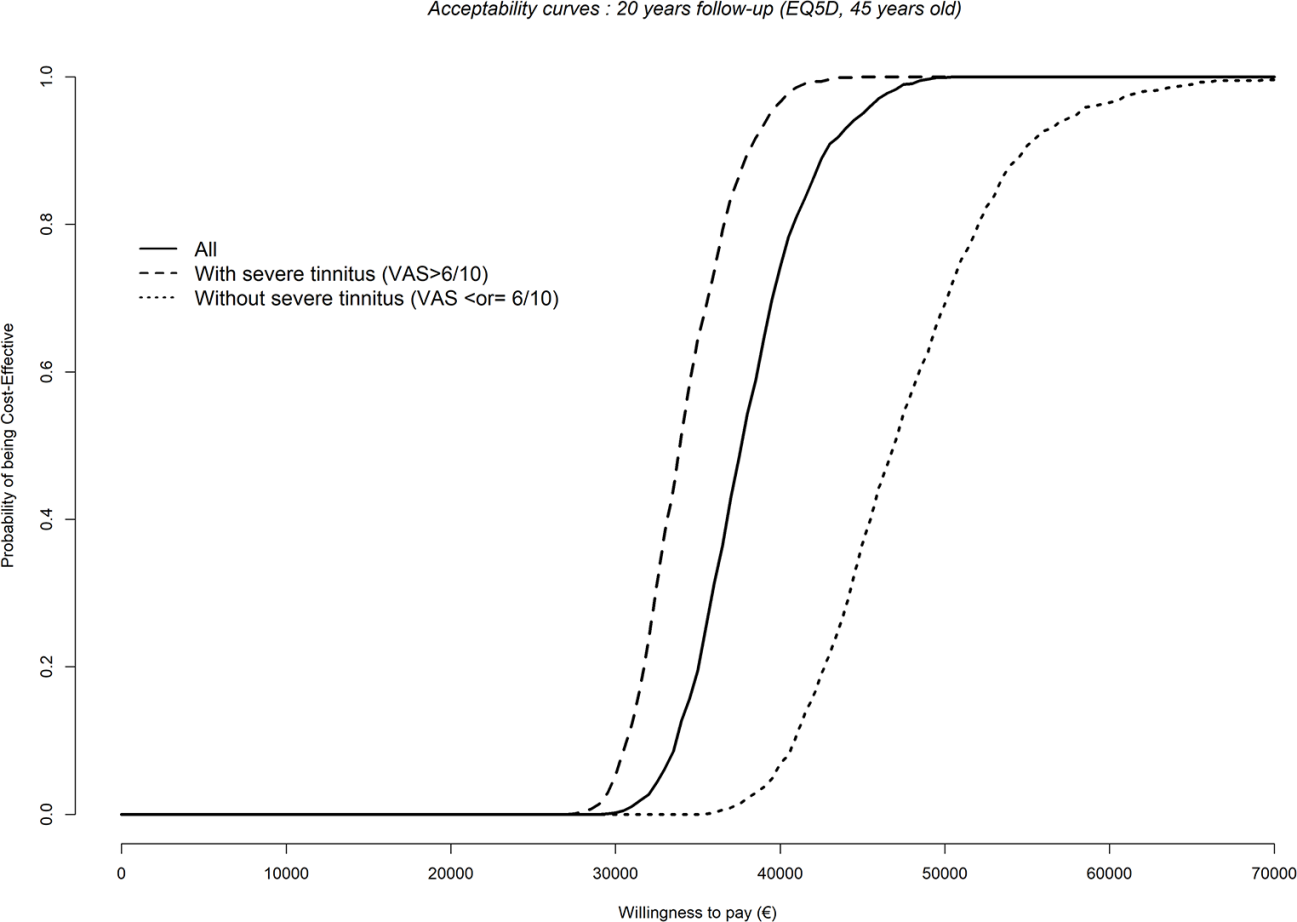
Acceptability curves at 20 years follow-up for all simulated subjects, or divided according to the presence or absence of a severe associated tinnitus (VAS > 6/10)

In the group of patients randomized for CI, additional cost-utility analyses were performed after HUI3 mapping, according to the presence of a severe tinnitus (> 6/10 on VAS; *n* = 13 in the CI arm, *n* = 15 in the initial observation arm; see Table 1). CI was more cost-effective in the participants with severe tinnitus with ICUR at 6-month follow-up of €229,268/QALY, versus €374,125/QALY in the patients without severe tinnitus. The acceptability curves (Fig. 3) highlight the higher probablity to be cost effective for the population of subjects with severe tinnitus. Using the Markovian model, ICUR in the subpopulation of patients with severe tinnitus ranged from €31,105/QALY at 10-year follow-up to €13,936/QALY at 50 years. In the population of patients without severe tinnitus, it was €50,750/QALY at 10-year follow-up and decreased to €22,954/QALY at 50 years.

## Discussion

To our knowledge, this is the first randomized clinical trial on cost-effectiveness of cochlear implantation in its more recent extended indication, i.e. SSD and AHL. At 6-month follow-up, and although utility was improved in CI arm, the ICUR was high, due to the significant cost of the medical device (€24,650) and the short duration of the clinical benefits it provided. However, when longer follow-up intervals were modeled, ICUR significantly decreased to reach between €57,561 /QALY at 10 years, €38,006/QALY at 20 years, down to €26,715 at 50 years. These ICURs were significantly lower when utility was measured using mapped HUI3 (see Table 1).

Recent considerations on ICUR acceptability suggest that the national gross domestic product (GDP)/inhabitant/year would be a relevant threshold of ICUR acceptability considering inter-country economic situation variability [29, 30]. This indicator should of course be interpreted in a context-specific healthcare system [31]. In France, GDP was €36,879/inhabitant for 2021 [32]. Therefore, CI could be considered as an efficient treatment in SSD/AHL after 10 to 20 years follow-up, according to the utility measurement which is considered. Such a result reflects the positive impact of CI on quality of life of patients with SSD or AHL. A recent meta-analysis identified 33 studies where a significant improvement of quality of life was reported after CI, using mainly Speech, Spatial and Quality of Hearing Questionnaire or Nijmegen Cochlear Implant Questionnaire [33]. Although this improvement was consistently found across studies, its magnitude could vary by a factor up to 10. Similarly, in the present population, Marx et al. [20] previously reported a significant impact of CI at 6-month follow-up, with an important interindividual heterogeneity.

In our series, CI would be particularly efficient in patients with associated incapacitating tinnitus, with a modelled ICUR of €31,105 (EQ-5D mapped HUI3) to €51,539 (EQ-5D)/QALY at 10-year follow-up. This converges with the results of the pioneering study by Van de Heyning et al. [34], and numerous successive reports [35, 36], which emphasized both the magnitude and the consistency of the effect of CI on severe tinnitus in the case of SSD/AHL. Indeed, a significant alleviation of tinnitus is reported in 69 to 86% of patients (see [13, 37] for review), with a complete suppression found in approximately 20% of subjects [13]. In addition, many reports, including systematic reviews with meta-analysis, have highlighted the positive average effect of CI on horizontal localization skills and speech recognition in challenging noisy environments [33, 35, 36]. Clinical and economic efficacy of CI in this specific population of SSD/AHL subjects support the validation of CI indications extension through reimbursement procedures. These results are line with ICURs extrapolated by Seebacher et al. [19] from utility measures obtained in 20 SSD patients who received a CI in Muigg et al. [38]. Using this Markovian model indeed, an ICUR of €34,862/QALY was found at 20 years after treatment. Likewise, Dreyfuss et al. [18] based their cost-utility analysis on clinical utility data obtained from previous studies, and included analyses where HUI3 scores were derived from the Speech, Spatial and Qualities of Hearing Questionnaire.

When compared with traditional unilateral CI indications, i.e. severe-to-profound bilateral hearing loss, CI utility appears reduced in SSD/AHL. Indeed, the mean improvement in utility reported using HUI3 by UK Cochlear Implant Study Group in 2004 [4] was 0.197 [0.176–0.218) where it was between 0.0459 (using EQ-5D) and 0.0945 (using Eq. 5D-HUI3 mapping) in the present study. Cost-utility was assessed as the net monetary benefit in the 2004 study and provided an incremental cost of €27,142 [24532–30323] per QALY gained, over the individual lifetime horizon. Given the 20 years which separate the two studies and the different methods used to calculate cost-utility indices, these comparisons should be considered with caution.

Nonetheless, it is probably more consistent to compare the incremental utility and cost-utility ratio of CI in SSD/AHL with the same parameters in the case of bilateral cochlear implantation for the second implant. Indeed, in those cases, a portion of the utility gain might reflect the improvement of quality of life related to the partial restoration of binaural hearing. Interestingly, Summerfield et al. [39] reported a quite comparable utility gain (0.03 after 9 months follow-up versus 0.0459 at 6 months in our study) for the second implant in bilateral CI in adults while this value reached 0.09 in the study of Chen et al. [40], still in line with our EQ-5D mapped HUI3 utility scores (0.0945). These values emphasize the relative but clinically significant utility of CI in SSD/AHL, as the second implant in the case of bilateral severe-to-profound hearing loss. Therefore, in countries where the second implant is funded for bilateral severe-to-profound deafness, these similarities advocate for the reimbursement of CI in SSD and AHL, at least in patients with accompanying severe tinnitus. Furthermore, the favorable cost-effectiveness for very long-term horizons also supports the coverage of CI for SSD in children, based on several efficacy demonstrations in this population [41–43].

The implementation of an MMS model inherently leads to a selection of hypotheses, regarding life expectancy or probabilities of transition between the different states. Here, it was notably hypothesized that, 1/our population have same life expectancy to the general population, 2/ cost and utility values are modeled for each state (assumptions described in supplementary file 1), 3/ complications may happen mainly during the first year following CI procedure, 4/ the odds of stopping using CI because of lack of effectiveness only occur during the first 2.5 years, 5/ patients with complication don’t have a second (i.e. rate negligible), 6/ probabilities of CI explantation are constant and are similar whatever the status of the patient (normal use, minor or major event states) 7/ there was no change in utility depending on the age or in the occurrence of minor or major event, explantation-reimplantation states. These assumptions were thought to provide a reliable approximation to reality. Indeed, they were made using the publications based on the French registry database on cochlear implantation activity [26, 27] which fit perfectly to our context.

### Limitations

The main limitation of this study is related to the short-term follow-up period (6 months) of the participants. This accounts for the very high initial ICUR values and emphasizes the need for models to assess the evolution of ICUR depending on the utility which has been measured.

The second limitation is related to the relatively low number of patients (51 in the group RCI) and the missing data which had to be imputed (6 to 8 per arm in the group RCI). Nevertheless, this number of SSD/AHL patients remains higher or in line with most studies on CI in SSD or AHL and the method used for data imputation is robust.

## Conclusion

This randomized controlled trial demonstrated that CI was an efficient treatment option in SSD and AHL, from follow-up at 10 to 20 years depending on hypothesis. This finding is particularly true for subjects with disabling associated tinnitus, who obtain most satisfactory results, in terms of treatment utility. Where possible, health care systems should thus cover this CI indication extension.

## Electronic supplementary material

Below is the link to the electronic supplementary material.

Supplementary Material 1

## Data Availability

The data that support the findings of this study are available on reasanable request from the corresponding author (MM).
