# Supplementary material for: Cost-utility of cochlear implantation in single-sided deafness and asymmetric hearing loss: results of a randomized controlled trial
Source: Eur J Health Econ. 2024 Nov 20;26(5):735–44. doi: 10.1007/s10198-024-01740-9 (PMC12204948; doi:10.1007/s10198-024-01740-9)

Supplementary file 1. Real-world costs during 6-months period according to Cochlear implant arm or control.

|  | CI arm | | | Control arm | Dif | P |  |  |
| --- | --- | --- | --- | --- | --- | --- | --- | --- |
|  | Mean  [95 Conf Int] | | | Mean  [95 Conf Int] | ∆IC-ABS |  |  |  |
| Inpatients stays* | 1699.68  [707.08; 4029.81] | | | 636.25  [247.77; 1748.71] | 1063.43 | 0.0048 |  |  |
| Consultations | 105.15  [64.03; 180] | | | 77.2  [46.7; 127.31] | 27.95 | 0.3518 |  |  |
| Medical acts | | | 187.49  [94.9; 359.31] | 95.96  [57.99; 202.33] | | 91.53 | 0.2607 | |
| Paramedical acts | 599.16  [248.07; 1796.83] | | | 77.92  [24.7; 179.78] | 521.24 | 0.0015 |  |  |
| Medications | 484.22  [84.23; 1186.71] | | | 124.21  [54.01; 387.96] | 360.01 | 0.1628 |  |  |
| Medical devices | 524.57  [87.15; 2565.73] | | | 96.14  [38.86; 207.33] | 428.43 | 0.0412 |  |  |
| Transports | 84.63  [29.67; 195.58] | | | 58.53  [4.17; 216.27] | 26.1 | 0.125 |  |  |
| Daily allowance | 285.55  [106.62; 745.9] | | | 354.65  [32.6; 1400.33] | -69.1 | 0.2423 |  |  |
| Global without CI | 4061.09  [1736.49; 10381.29] | | | 1584.74  [774.53; 3191.4] | 2476.35 | 0.0035 |  |  |
| CI | 24650 | | | 0 |  |  |  |  |
| Global with CI | 28711.1  [26386.5; 35031.3] | | |  | 27798.3 |  |  |  |

CI: Cochlear implant; *Do not consider impatient costs related to implantation of CI

Supplementary file 2. The HUI3 mapping procedure

The mapping procedure transforms utility values coming from one questionnaire (here EQ-5D) to utility values coming from another (here HUI3) [1]. All responses to the HUI3 and EQ5D questionnaire dimensions are needed to proceed to the mapping. Once all responses are gathered, the mapping procedure consists to 1/regress the HUI3 utilities values using EQ5D questionnaire dimensions as in the Abdin et al cohort, adjusted with age and gender then 2/ to apply coefficient estimated to EQ5D dimensions in our cohort.

We thank Dr Edimansyah Abdin and his team for sharing their data [2] and for detailing age and gender of patients in their population. It is to note that their population was affected with psychiatric disorders (depressive symptoms, n=249; and schizophrenia, n=251). To ensure the correct coefficient estimate we have first validated that EQ5D-utility distribution was similar in both our population as shown below. We have used the entire population in our cohort meaning the BAHA CROS and abstention population with no missing data at baseline and 6 months after (n=130).


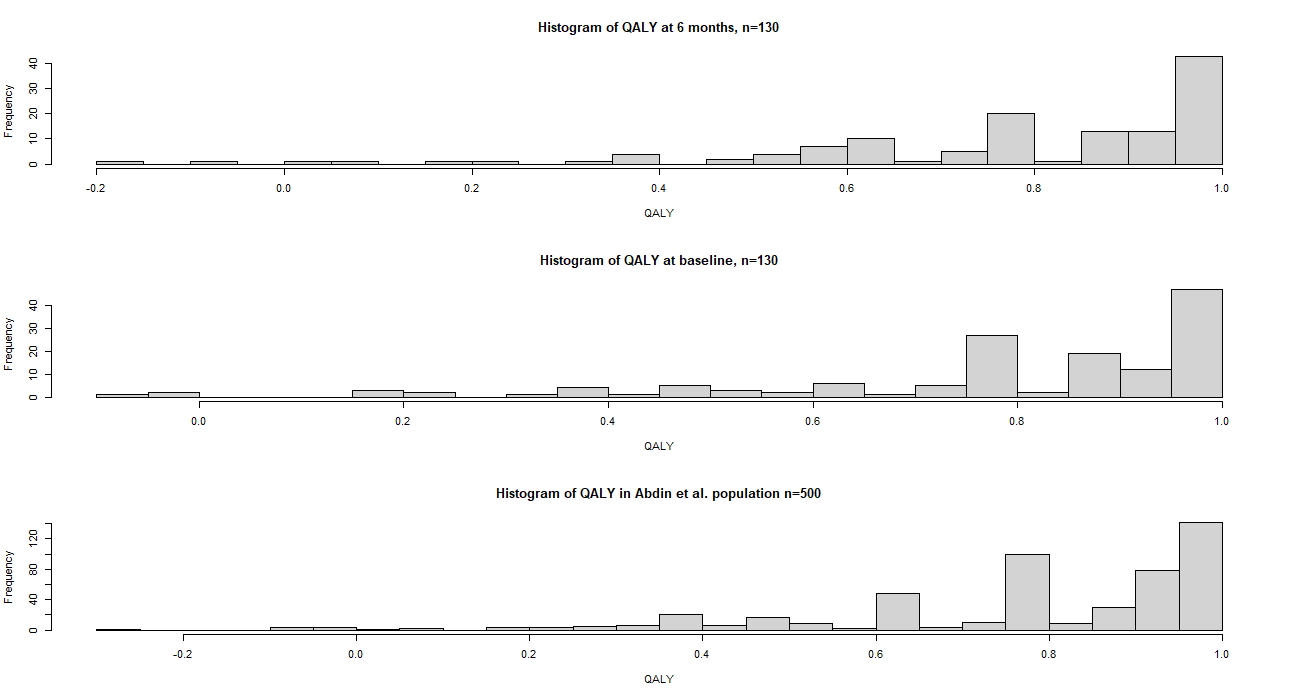


We assumed that distribution was comparable enough to proceed to the mapping procedure.

First HUI3 utility values were re-estimated using French preference-based values. Negative values were replaced by a value of 0.01 and a beta regression model was implemented to obtain the coefficients shown below.

Beta model used to predict HUI3 utility value given EQ5D dimension, age and gender

|  | Estimate | Std. | Error | z | value |
| --- | --- | --- | --- | --- | --- |
| (Intercept) | 3.187242 | 0.285678 | 11.157 | 2.00E-16 | *** |
| age | 0.000721 | 0.0041 | 0.176 | 0.860329 |  |
| Female :Male | -0.18812 | 0.08593 | -2.189 | 0.028583 | * |
| EQ5D3L_mobility | -0.56013 | 0.153561 | -3.648 | 0.000265 | *** |
| EQ5D3L_selfcare | 0.036092 | 0.220869 | 0.163 | 0.870197 |  |
| EQ5D3L_usualactivities | -0.31436 | 0.09641 | -3.261 | 0.001112 | ** |
| EQ5D3L_anxietydepression | -0.57714 | 0.081295 | -7.099 | 1.25E-12 | *** |
| EQ5D3L_paindiscomfort | -0.44958 | 0.089067 | -5.048 | 4.47E-07 | *** |

Finally, coefficients were calculated using the following equation for $i$ patients :

$$HUI3_{i}\sim Age_{i}+Sexe_{i}+ EQ5D3L_{mobility, i}+ EQ5D3L_{selfcare, i}+ EQ5D3L_{usual activities, i}+ EQ5D3L_{anxiety/depression,i}+EQ5D3L_{pain/discomfort, i}$$

1. Young TA, Mukuria C, Rowen D, Brazier JE, Longworth L. Mapping Functions in Health-Related Quality of Life: Mapping from Two Cancer-Specific Health-Related Quality-of-Life Instruments to EQ-5D-3L. Med Decis Making. 2015 Oct;35(7):912-26. doi: 10.1177/0272989X15587497. Epub 2015 May 21. PMID: 25997920; PMCID: PMC4574084.
2. Abdin E, Chong SA, Seow E, Tan KB, Subramaniam M. Mapping the PHQ-8 to EQ-5D, HUI3 and SF6D in patients with depression. BMC Psychiatry. 2021 Sep 13;21(1):451. doi: 10.1186/s12888-021-03463-0. PMID: 34517871; PMCID: PMC8438835.

Supplementary file 3. Multi state modeling : details on the main assumption

A Markovian Multi-state transition model was used to assess the ICUR of CI on a lifetime horizon. The time step was set up to 6-months, to fit with the interval of our data for the primary outcome.

Two models were taken into account: one model considering SSD/AHL patients after CI, and one model considering not implanted SSD/AHL patients in order to compare outcomes (i.e. costs & QALY) and build the ICUR evolution .

A patient with a CI starts with a cost related to the implantation. Following this initiation phase, a patient may belong to the following states : $CI-USERS| Normal use$, $CI-USERS| Minor Event$, $CI-USERS| Major Event$, $CI-USERS| Explantation/Reimplantation$, $CI NON-USERS$ or to the $DEATH$ state. All transition probabilities are described in e-Table 1.

*Description of all states and possible path.*

A patient in $CI-USERS| Normal use$ could remain in this state or transit to $CI NON-USERS$ state only during the first 3-time steps following an implantation. This transition may occur when the CI does not provide enough efficacy and when the patient prefers to stop use the CI. Nevertheless, in the vast majority of cases, these patients are not explanted. Therefore, no extra cost for explantation is incurred but utility in these patients becomes similar to that of the patients who are not implanted, i.e.$NON-CI$ utility. Patients among $CI NON-USERS$ remain in this state in the entire follow-up (exception one case, see below).

In addition, a patient could transit from $CI-USERS| Normal use$ to $CI-USERS| Minor Event$ or $CI-USERS| Major Event$ only in the two first time steps following an implantation, which correspond to a one-year period with a similar probability for the two time steps. Minor or major events are those described in the EPIIC study. These states are associated with extra costs to deal with the event but we have hypothesized no impact on utility because these kinds of event are usually monitored during short-term periods. Besides, the impact on utility, if existing, is unmeasurable here. A patient in $CI-USERS| Minor Event$ or $CI-USERS| Major Event$ states may come back in $CI-USERS| Normal use$ or go to $CI-USERS| Explantation/Reimplantation$state as patient in $CI-USERS| Normal use$ state. The three transitions probabilities to $CI-USERS| Explantation/Reimplantation$ were assessed using French National registry for cochlear implantation data.

Patients in $CI-USERS| Explantation/Reimplantation$ account for important extra costs due to explantation and reimplantation. Average of 86% of patients then transit in the $CI-USERS| Normal use$ but it has been assessed that 14% are explanted directly but implanted only at the next time step. These 14% then go to the $CI NON-USERS$ before coming back to the $CI-USERS| Normal use$ state. The costs associated with the $CI-USERS| Explantation/Reimplantation$ state is considered when the patient transit in this state despite he is re-implanted the next time step.

The age-dependent probability of death is inputted in each transition and was extracted from the French National Institute of Statistics and Economic Studies. We then hypothesized similar life expectancy of between general population and our study population. We have considered that the sum of all probabilities associated with a state is equal to 1. In this context, all probabilities firstly assessed were decreased according to the death probability.

We have not considered the probability to transit from $CI-USERS| Minor Event$ or $CI-USERS| Major Event$ to $CINON-USERS$ because the proportion of patients in both situations is too low to be determined (i.e. occurring only in start of management).

The second model involves the patients who are not implanted, i.e. the $NON-CI$state. Patients in this state remain in this state or transit to the death state. In the same way, 1000 iterations of 1000 patients are included in this model with similar characteristics regarding age and death probabilities.

At each time step, the cost and utility measurements according to the state were conducted. These measurements mainly relied on our data, on the French framework of the hospital activity pricing and expert opinion to assess which health consumption should be considered. Two analyses were implemented using utility estimated by EQ5D QoL or the HUI3 mapped QoL.

*Scenarios*

Basecase: Cohorts of 1000 patients, aged of 45 years old. Simulations were implemented for 10 to 50 years of time horizon.

Sensitivity analyses: All these analyses were implemented for 10 to 50 years of time horizon. The different scenarios depended on: the age of the population (20, 40 and 60 years old), the presence or absence or a severe associated tinnitus (VAS>6/10; i.e. different utilities values but similar costs of management) and the mapped HUI3 utility instead of our EQ-5D data.

*Additional hypotheses*

We hypothesized homogeneous transition probability (excepted those noted above: $CI-USERS| Normal use$ to $CI-USERS| Minor Event$ or $CI-USERS| Major Event$), states cost and utility during the entire simulation follow-up. A 2.5% actualization rate was used during the first 30 years and switched to 1.5% after 30 years. It was also hypothesized that patient with a minor or major event only presented one minor or major event.

The total cost and utility between CI model and NON-CI model were subtracted to assess differential cost, differential utility and the Incremental cost utility ratio after CI. Confidence ellipses were built for each scenario but only the most significant (see figure 2 for basecase using EQ-5D, see supplementary file 2 for basecase using mapped HUI3).

e-Table 1. Main model parameters with reference used

|  |  | Value | Comment | Reference |
| --- | --- | --- | --- | --- |
| Cost | IC USERS : initiation (0) | C(0): Processor: €6,000 + Implant: €13,650 + Hospitalization: €5,000 | Only once and not applied to comparison group | French National Health Insurance (FNIH) |
|  | IC USERS \| Normal use (1) | C(1): Battery package: €60 + ENT consultations: €30 | + €6,000 every 5.5 years for replacement of external processor | FNIH (valorization) & Experts (type and quantity) |
|  | IC USERS \| Major event (2) | C(2): C(1) + Hospitalization (DRG 03M15Z or 03M15Y) : mean (sd) = €1411 (1034) ==> Gamma(Shape,Scale) = Gamma(1.859983, 758.6089) | Both CIM-10 code used that is why we averaged them costs | HITA & Experts |
|  | IC USERS \| Minor event (3) | C(3): C(1) + ENT consultation : €30 + (AMOXICILLINE : €4.51 (10% of patients) or  (TANGANIL : €4.25 + PRIMPERAN : €2.28 (20% of patients)) or  (videonystagmoscopy: €57.60 + TANGANIL : €4.25 + PRIMPERAN : €2.28 (70% of patients))) | Three situations identified with associated rate, randomly draw. | FNIH & Experts |
|  | IC USERS \| Explant / Re-implant (4) | C(4) : C(1) + C(0) |  | FNIH & Experts |
|  | IC NON-USERS (5) | ENT consultation: €30 | + Explantation : €1968.50, only one time when transiting from (4) to (5) | FNIH |
|  | NON-IC (6) | ORL consultation: €30 every year ==> €15 each time step |  | FNIH |
|  | Death (7) | 0 |  |  |
| Utility | IC USERS : initiation (0) | NA |  | NA |
|  | IC USERS \| Normal use (1) | U(1) : Mean (sd) = 0.8423 (0.18) ==> Beta(α,β) = Beta(2.610888, 0.4888248) U'(1) : Mean (sd) = 0.6943 (0.15) ==> Beta(α,β) = Beta (5.854473, 2.575294) | Beta parameters identified through mean and var | Our data without mapping procedure (U1) or with mapping U'(1) |
|  | IC USERS \| Major event (2) | U(1) or U'(1) | Expert hypothesis: no alteration on Utility |  |
|  | IC USERS \| Minor event (3) | U(1) or U'(1) | Expert hypothesis: no alteration on Utility |  |
|  | IC USERS \| Explant / Re-implant (4) | U(1) or U'(1) | Expert hypothesis: no alteration on Utility |  |
|  | IC NON-USERS (5) | U(2) : Mean (sd) = 0.78 (0.18) ==> Beta(α,β) : U(2) = Beta(3.351111, 0.9451852) U'(2) : Mean (sd) = 0.5998 (0.15) ==> Beta(α,β) : U'(2) = Beta (5.799132, 3.869311) |  | Our data without mapping procedure U(2) or with mapping U'(2) |
|  | NON-IC (6) | U(2) or U'(2) |  |  |
|  | Death (7) | 0 |  |  |
| Transition | (1) ==> (2) | 2.04% [1.6 ; 2.6] on 3483 implants ==> Beta(α,β) =Beta(71.0328,3410.97) | Only the year after an implantation | [1] |
|  | (1) ==> (3) | 3.47% [2.9 ; 4.1] on 3483 implants ==> Beta(α,β) = Beta(120.8254, 3361.175) | Only the year after an implantation | [1] |
|  | (1) ==> (4) | 0.232% on 3178 implants ==> Beta(α,β) = Beta(7.37064,3169.63) |  | [2] |
|  | (2) ==> (1) | 1- [(1) ==> (2)] ==> Beta(α,β) = Beta(**3169.63, 7.37064)** |  | [2] |
|  | (3) ==> (1) | 1- [(1) ==> (2)] ==> Beta(α,β) = Beta(**3169.63, 7.37064)** |  | [1] |
|  | (2) ==> (4) | Similar to (1) ==> (4) : Beta(α,β) = Beta(7.37064,3169.63) |  | [2] |
|  | (3) ==> (4) | Similar to (1) ==> (4) : Beta(α,β) = Beta(7.37064,3169.63) |  | [2] |
|  | (4) ==> (1) | 86% on 37 re-implantation ==> Beta(α,β) = Beta(30.96,5.04))) |  | [2] |
|  | (4) ==> (5) ==> (1) | 14% on 37 re-implantation next time step ==> Beta(α,β) = Beta(5.04, 30.96))) then 100% of them transite to(1) | One time step delay for 14% of patients | [2] |
|  | All ==> (7) | National data : probability depending on age. | Applied on each probability | [3] |
|  | (1), (2), (3) ==> (5) | Tavoiera : 4.4% genrally 6 months / Marx (Ref!?) : 20% generally 2.5 years ==> 12% under 1.5 years ==> 4% on 3 time step | Averaged of 2 study | [4, 5] |

References supplementary file 3 / e-Table 1.

1. Parent V, Codet M, Aubry K, et al. The French Cochlear Implant Registry (EPIIC): Cochlear implantation complications. *Eur Ann Otorhinolaryngol Head Neck Dis*. 2020;137 Suppl 1:S37-S43. doi:10.1016/j.anorl.2020.07.007
2. Hermann R, Coudert A, Aubry K, et al. The French National Cochlear Implant Registry (EPIIC): Cochlear explantation and reimplantation. *Eur Ann Otorhinolaryngol Head Neck Dis*. 2020;137 Suppl 1:S45-S49. doi:10.1016/j.anorl.2020.07.006
3. National Institute of Statistics and Economic Studies - vital statistics and population estimates. Available 06/2023 : <https://www.insee.fr/fr/statistiques/4503155?sommaire=4503178#titre-bloc-5>
4. Távora-Vieira D, Acharya A, Rajan GP. What can we learn from adult cochlear implant recipients with single-sided deafness who became elective non-users?. *Cochlear Implants Int*. 2020;21(4):220-227. doi:10.1080/14670100.2020.1733746
5. Marx M, Mosnier I, Vincent C, Bonne NX, Bakhos D, Lescanne E, Flament J, Bernardeschi D, Sterkers O, Fraysse B, Lepage B, Godey B, Schmerber S, Uziel A, Mondain M, Venail F, Deguine O. Treatment choice in single-sided deafness and asymmetric hearing loss. A prospective, multicentre cohort study on 155 patients. Clin Otolaryngol. 2021 Jul;46(4):736-743. doi: 10.1111/coa.13672.


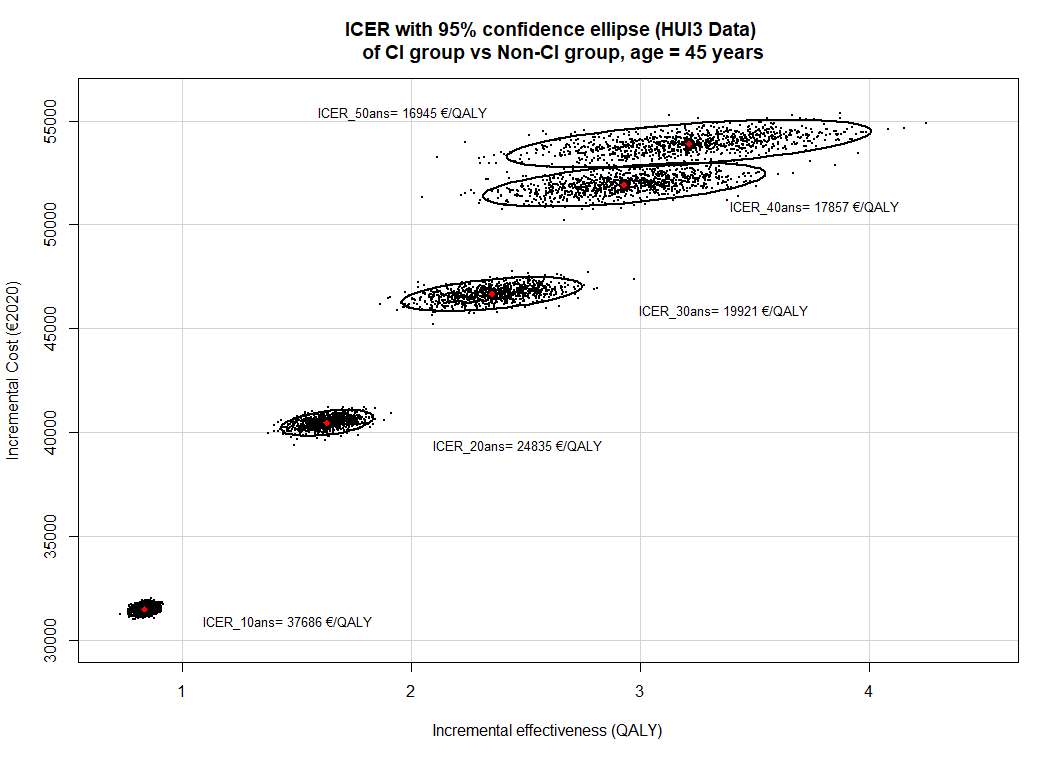
Supplementary file 4. Confidence ellipses using HUI3 mapped utility

Supplementary file 5. Flowchart of the study


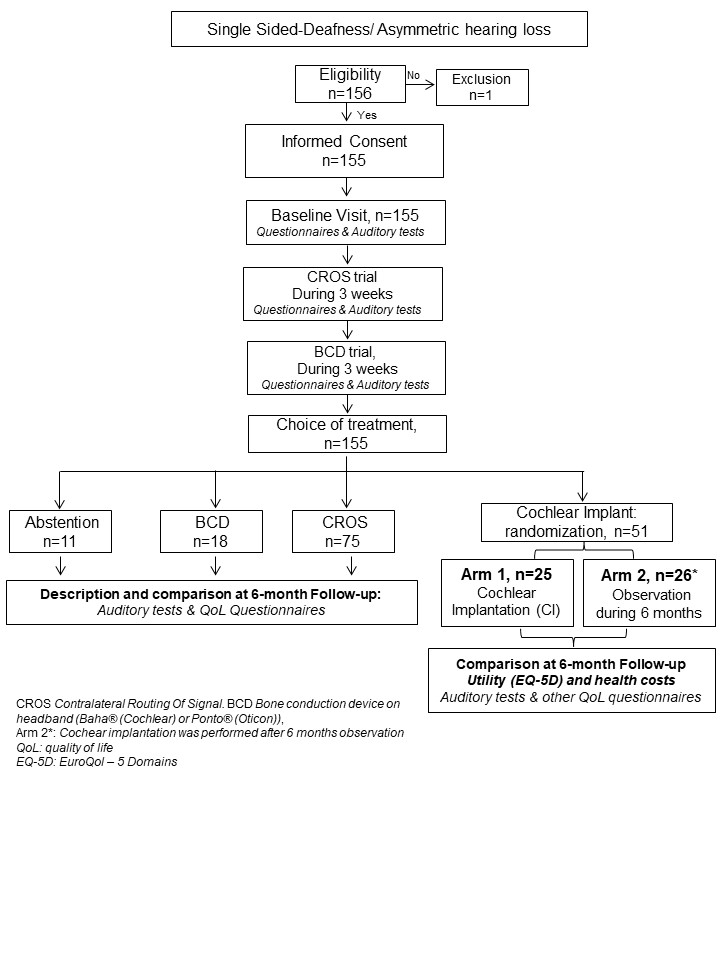

Supplement: Supplementary file 1 — Supplementary Material 1 [file 10198_2024_1740_MOESM1_ESM.docx]
